# A dimensional link between gain–loss economic preferences and psychopathology

**DOI:** 10.64898/2026.09.09.750300

**Published:** 2026-09-15

**Authors:** Yang-Yang Feng, Ilya E. Monosov

## Abstract

Human decision-makers differ in how they value and trade off reward, risk, and information when evaluating future gains and losses, and these preferences are central to well-being. However, how multi-attribute economic valuation relates to psychopathology remains unclear. Here, 1,954 individuals completed a transdiagnostic symptom battery and a multi-attribute decision-making task that independently manipulated expected reward, reward uncertainty, and non-instrumental information under matched gain and loss contexts. On average, when choosing among gains, participants preferred larger rewards, avoided risk, and sought advance information. When choosing among losses, they still preferred better outcomes and advance information, but less strongly. Their risk preference, by contrast, reversed, from risk-averse to risk-seeking. Substantial individual variability accompanied these population-level context effects, and different patterns of variability were associated with separate dimensions of psychopathology. Anxious– depressive and social withdrawal symptoms were associated with increased reward sensitivity across contexts, whereas compulsive–intrusive symptoms were associated with reduced reward sensitivity. Symptom-related differences in risk and information preferences were not fully explained by a uniform scaling of overall economic sensitivity, revealing additional attribute- and context-specific variation. A data-driven analysis summarized these symptom–preference associations in a single latent dimension that contrasted compulsive–intrusive symptoms with anxious–depressive and social withdrawal symptoms. An independently identified subgroup that strongly sought information about gains but avoided it about losses mirrored this profile and was enriched for compulsive–intrusive symptoms. These findings reveal structured, low-dimensional variation linking multi-attribute decision-making to psychopathology and could support a dimensional, task-based approach to psychiatric nosology.

## Full Text Availability

The license terms selected by the author(s) for this preprint version do not permit archiving in PMC. The full text is available from the preprint server.

